# PTPRS drives adaptive resistance to MEK/ERK inhibitors through SRC

**DOI:** 10.18632/oncotarget.27335

**Published:** 2019-11-26

**Authors:** Thomas B. Davis, Mingli Yang, Heiman Wang, Changgong Lee, Timothy J. Yeatman, W. Jack Pledger

**Affiliations:** ^1^Department of Surgery, University of Utah, Salt Lake City, UT 84132, USA; ^2^Gibbs Cancer Center & Research Institute, Spartanburg, SC 29303, USA; ^3^Cell Response and Regulation Program, Huntsman Cancer Institute, University of Utah, Salt Lake City, UT 84132, USA; ^4^Oncology Clinical Program, Intermountain Healthcare, Murray, UT 84107, USA

**Keywords:** colorectal cancer, PTPRS, ERK, SRC, adaptive resistance

## Abstract

PTPRS is the most commonly mutated receptor tyrosine phosphatase in colorectal cancer (CRC). PTPRS has been shown to directly affect ERK and regulate its activation and nuclear localization. Here we identify that PTPRS may play a significant role in developing adaptive resistance to MEK/ERK inhibitors (MEKi/ERKi) through SRC activation. Moreover, we demonstrate a new clinical approach to averting adaptive resistance through the use of the SRC inhibitor, dasatinib. Our data suggest the potential for dasatinib to enhance the efficacy of MEKi and ERKi by preventing adaptive resistance pathways operating through SRC.

## INTRODUCTION

Colorectal Cancer (CRC) is one of the leading causes of cancer deaths in the USA which could be surgically cured with early detection; however, many CRC tumors are not diagnosed until late stage when cure rates are low [1]. While 80% of CRC tumors have an altered WNT pathway, many CRC tumors (50%) also have activated KRAS/BRAF, and these tumors are associated with poor clinical outcomes [2–7]. Effective inhibitors of MEK, ERK and AKT are available, although they have proven ineffective for most CRC, perhaps due to intrinsic or adaptive resistance [8–13]. It has been previously noted that SRC activity is elevated in many CRC tumors, and thus could play a role in generating resistance to therapies [14–17]. Moreover, recent investigations have pointed out that adaptive resistance to MEK inhibitors in ovarian cancer cell lines was due to elevation of activated SRC [16].

We recently discovered that protein tyrosine phosphatase receptor S (PTPRS) was the most frequently mutated gene (~10%) in the family of protein tyrosine phosphatases in our CRC tumor collection (*n* = 468); similar results were reported for the Dana Farber CRC database [5, 18]. These mutations correlated with an increase in a validated 18-gene RAS pathway signature score, signifying an increase in RAS pathway signaling [18–20]. Moreover, we showed that the loss of PTPRS activity in CRC cell lines brought about increased ERK activation [18]. Most of the native mutations we found in human CRC were missense mutations located throughout the PTPRS coding region including the carboxyl terminal end, the transmembrane regions, the activity domain and the amino-terminal region [18]. We verified that many of the native missense mutations in PTPRS brought about a reduction in its phosphatase activity as measured by the dephosphorylation of tyrosine phosphorylated ERK [18].

PTPRS has been shown to have a role in neural system biology, spinal injury repair [21–23], intestinal permeability, ulcerative colitis, autophagy [24–26] and tumor suppression [27, 28]. PTPRS has also been postulated to act as a metastatic suppressor and shown to have reduced expression in 80% of hepatocellular carcinoma (HCC) [29]. PTPRS promoter methylation was detected in HCC tumor samples and in HCC tumor cell lines [29]. Furthermore, PTPRS was shown to dephosphorylate EGFR in A431 cells, and genomic analysis revealed frequent mutations of PTPRS in head and neck cancer [30, 31]. Recently, we demonstrated a direct physical association of PTPRS and ERK, with the dephosphorylation of ERK preventing its activation and nuclear localization [18]. When PTPRS is knocked out (KO) using CRISPR in HCT116, a commonly used CRC model cell line, the phosphorylation of ERK was increased along with an increased phosphorylation of AKT [18]. Since the loss of PTPRS activity brought about an increased ERK and AKT phosphorylation in HCT116 KO cells without PTPRS activity, we were surprised to find that these KO cells were more sensitive to MEK/ERK inhibitors (MEKi/ERKi) than parental cells with PTPRS. Here we explore the mechanism whereby the loss of PTPRS activity induces increased drug response. Our data have led us to hypothesize that CRC cells without PTPRS are more sensitive to MEK or ERK inhibition because, unlike the parental cells, they cannot invoke an adaptive resistance response that bypasses MEK/ERK drug blockade. We investigated a possible role for SRC in therapeutic resistance to MEKi and ERKi using multiple genetic modifications of the HCT116 CRC cell line model. We now hypothesize that SRC activation is dependent on PTPRS, and is likely responsible for adaptive resistance to MEKi/ERKi.

## RESULTS

### The loss of PTPRS activity increased growth potential in CRC cell lines

**Figure 1:**
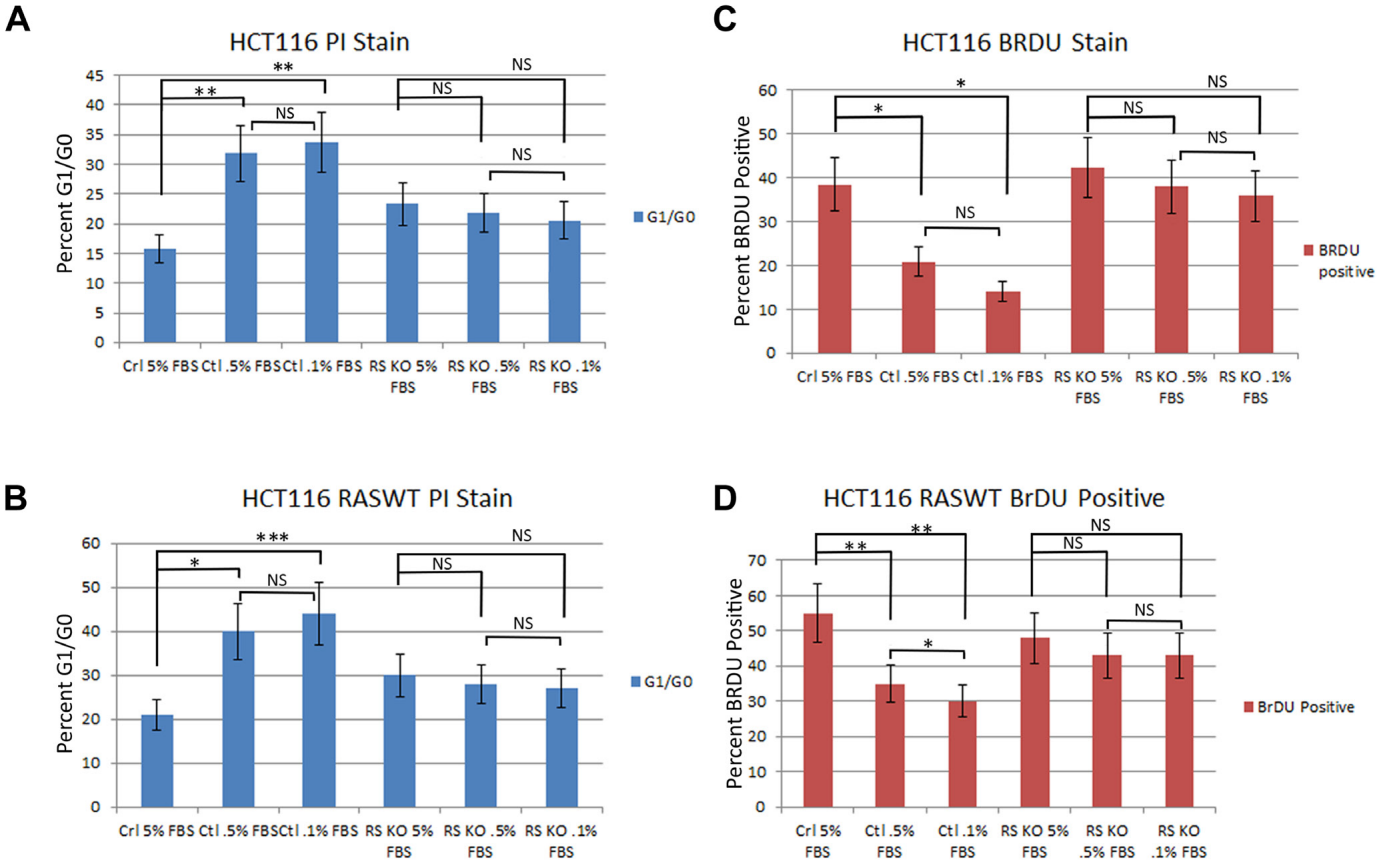
Knockout of PTPRS in KRAS mutant and KRAS WT cells increased growth potential. Parental HCT116 cells (mut KRAS) +/– PTPRS were cultured for 24 hours in RPMI media with 5.0%, 0.5% or 0.1% FBS. (**A**) HCT116 parental cells were stained with PI and (**C**) HCT116 cells in the same conditions for 24 hours were cultured with Brdu for 2 hours in order to compare the effects on DNA synthesis that occurred when serum was reduced. The parental cells with PTPRS activity cultured in low serum concentrations demonstrated a reduction in growth (PI analysis, Brdu assay) while the HCT116 PTPRS knockout cells maintained growth. The HCT116 cells with WT KRAS (+/– PTPRS) were cultured in the same serum concentrations as parental lines for 24 hours. Cells with WT KRAS were harvested and (**B**) stained for PI or (**D**) cultured 2 hours with Brdu. Like the parental HCT 116 cells, the WT RAS HCT116 cells showed a decrease in growth for cells with PTPRS expression, but not in cells lacking PTPRS activity. The paired, two-tailed *t* test was performed for comparisons. NS – not significant; ^*^ *p* < 0.05; ^**^ *p* < 0.01; ^***^ *p* < 0.001.

The loss of PTPRS activity in CRC cell lines produced increased ERK and AKT phosphorylation and increased downstream ERK signaling [18], therefore we sought to determine if the loss of PTPRS activity could produce an increased growth potential in cells with activated KRAS or with wild type (WT) RAS. We constructed with CRISPR an isogenically-paired CRC HCT116 MUT KRAS cell line +/– PTPRS [18]. Furthermore, paired cell lines (+/– PTPRS) were also made in isogenic HCT116 cells with wild type (WT) KRAS [18]. The paired cells(+/– PTPRS) each with WT or MUT KRAS were grown for 24 hours in culture medium with serum concentrations of 5.0%, 0.5% or 0.1% FBS. Cultures were harvested and stained with PI to determine cell cycle distribution. An increased number of cells in G1 phase (2N DNA) indicated a reduction in cells traversing the cell cycle and thus limited growth. As can be seen in Figure 1A and 1B, our analysis revealed that the CRC cells containing PTPRS, even with active (mutant) KRAS, showed an increase in the number of cells locked in the G1 phase (decreased growth) after a 24 hours culture period in low serum when compared to cells without PTPRS. Cells cultured in 5% FCS had fewer cells (20% less) stopped in G1 after 24 hours than cells in cultured in 0.5% or 0.1% FCS showing their serum requirement. The absence of PTPRS activity in cells with or without mutationally- activated KRAS produced less dependency on serum (Figure 1). In addition, we also observed decreased numbers of cells in active S phase (from 40–50% to 25–12%) in cells with PTPRS during a two-hour incubation with Brdu after 24 hours in cultured growth medium supplemented with low serum (0.1–0.5% FCS) (Figure 1C and 1D). This decrease was also observed in cells with KRAS activation. However, PTPRS KO cells had less serum dependency; more cells synthesized incorporating Brdu in low serum (0.1–0.5%) than cells with PTPRS activity with or without activated KRAS. Thus, KO cells without PTPRS had a lower requirement for serum, demonstrating higher growth potential, even in the presence of activated KRAS.

### Loss of PTPRS activity brought about increased AKT phosphorylation which is dependent on ERK activity

**Figure 2:**
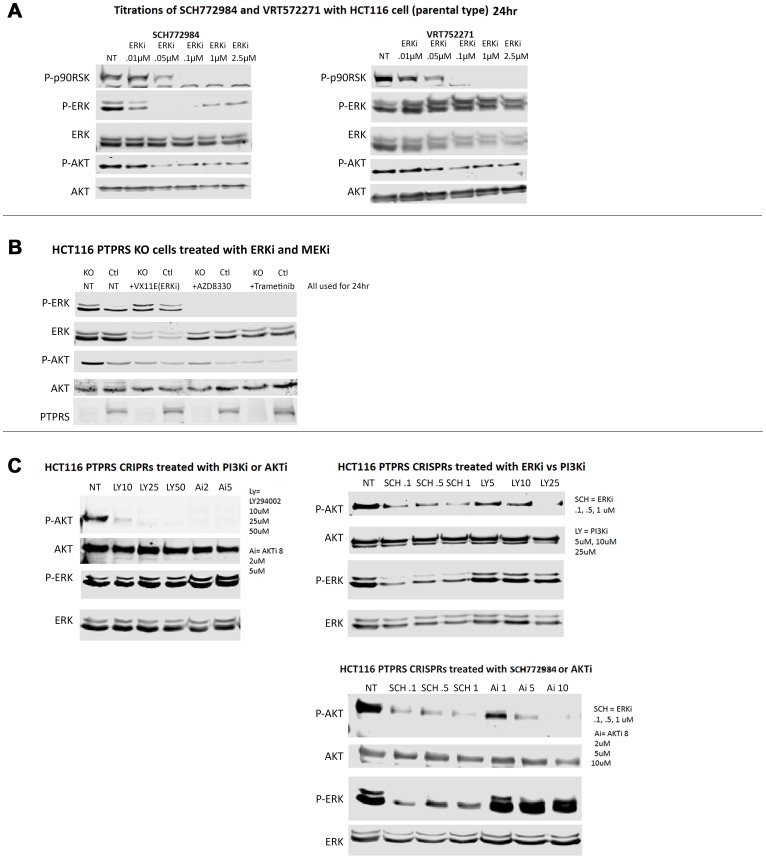
ERK and MEK inhibition decreased AKT-S473 phosphorylation but PI3K and AKT inhibition lacked effect on ERK phosphorylation. (**A**) Western blot analysis was performed on HCT116 cells treated for 24 hours with various concentrations of ERK inhibitors (SCH772984 (left) and VRT752271 (right)). Both inhibitors showed inhibition of ERK activity as seen by decreased phospho-p90RSK activation. Both inhibitors also decreased phospho-AKT-S473 levels. (**B**) HCT116 parental cells and PTPRS KO cells treated with ERK and MEK inhibitors demonstrated reduction of phospho-ERK1/2-T202/Y204 and reduced phospho-AKT-S473. (**C**) PI3Ki (LY294002) and AKTi (AKTi 8) completely reduced phospho-AKT-S473 levels, but had limited effects on phospho-ERK1/2-T202/Y204 in HCT116 parental cells and with CRISPR KO of PTPRS. Each experiment was performed three times. Quantitative densitometry analysis of each blot is available in Supplementary Figure 2.

We had previously shown that the loss of PTPRS activity in CRC cell lines brought about increased ERK and AKT phosphorylation [18]. In addition, and as expected this increased phosphorylation of ERK produced increased ERK signal transduction in downstream targets [18]. We sought to determine the effects of ERK inhibitors and noticed that cell cultures treated with inhibitors of ERK showed reduced phosphorylation of AKT (Figure 2A). The reduction in AKT phosphorylation was observed using several different ERK inhibitors. SCH772984 reduced the activity of ERK as can be seen by the inhibition of pERK and p-p90RSK. However, a second ERK inhibitor, VRT572271, was noticed to decreased the phosphorylation of p90RSK while ERK remained phosphorylated (Figure 2A). The decreased in the phosphorylation of AKT after ERK inhibition was not dependent on the presence or absence of PTPRS (Figure 2B). In addition, MEK inhibitors also brought about reduced ERK phosphorylation and reduced AKT phosphorylation (Figure 2A). On the other hand, when similar cultures were treated with either inhibitors of AKT or PI3K there was no reduction, or only a limited reduction, of the phosphorylation of ERK as compared to the untreated cells (Figure 2C). These data suggest that ERK activity is required for the increased AKT phosphorylation/activation or the stability of its activated state.

**Figure 3:**
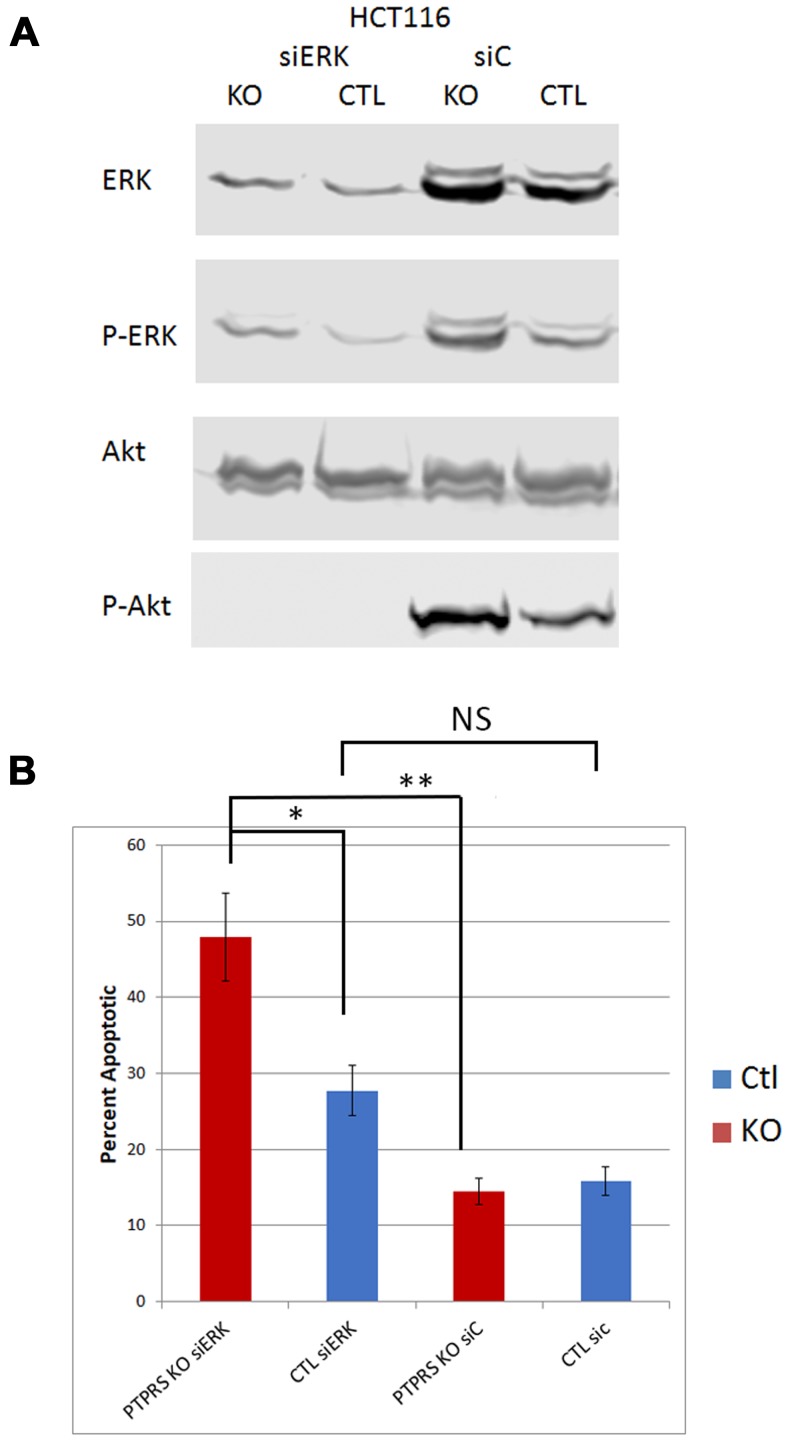
siRNA knockdown of ERK reduced phospho-AKT-S473. (**A**) Western blot analysis of parental HCT116 cells and PTPRS KO HCT116 cells treated for 48 hours with siRNA. Cells (PTPRS +/–) were treated with either a control siRNA or siRNA for ERK for 48 hours. All cells treated with siRNA for ERK had reduction in phospho-ERK and phospho-AKT-S473. Quantitative densitometry analysis is available in Supplementary Figure 3. (**B**) siRNA Knockdown of ERK induced apoptosis. Annexin V apoptosis assay following knockdown of ERK in HCT116 +/– PTPRS. Control siRNA and ERK targeted siRNA were transfected into both control and PTPRS KO HCT116 cells. Elevated apoptosis was seen in the PTPRS KO cells when ERK was knocked down. The paired, two-tailed *t* test was performed for comparisons. NS – not significant; ^*^ *p* < 0.05; ^**^ *p* < 0.01.

Since the inhibition of ERK via chemical inhibitors diminished the induced level of AKT phosphorylation, we used siRNA to ERK to ensure the specificity of an ERK activity requirement for the increased AKT phosphorylation. CRC cells were treated with siRNA to ERK; Figure 3A shows a decrease in total ERK. Furthermore, our data also showed the decrease in phosphorylation of ERK and of AKT in cultures with siRNA ERK knock-down. siRNA knockdown of ERK consistently produced a reduction in AKT phosphorylation greater than that observed when cells were treated with ERK inhibitors (Figure 3A).

### The loss of PTPRS activity gave rise to an increased apoptotic response to ERK inhibition

**Figure 4:**
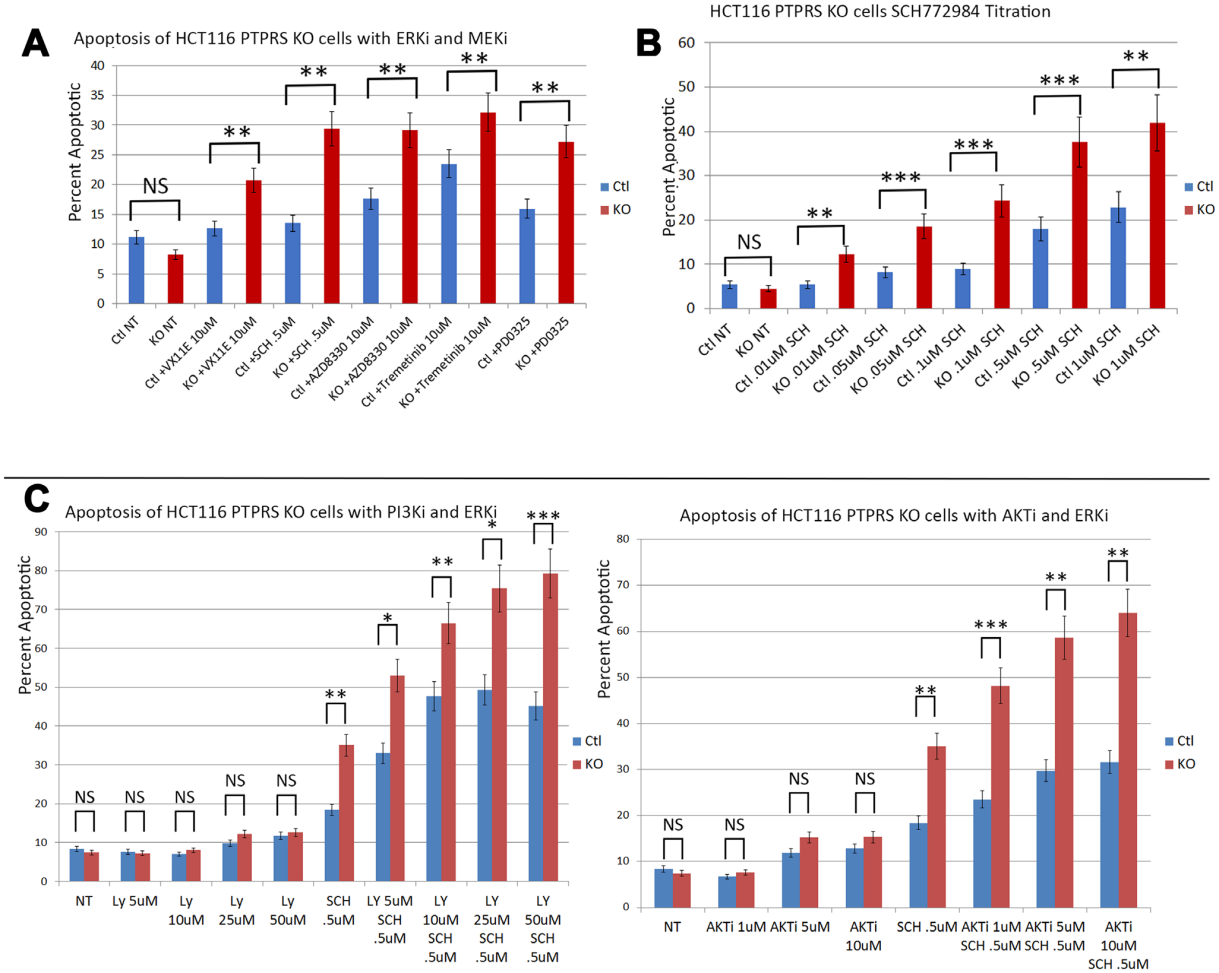
PTPRS KO cells produced increased apoptosis in response to ERK inhibition. Annexin V assays demonstrated that HCT116 cells with PTPRS KO had increased sensitivity to ERK inhibition. (**A**) Annexin V flow assay for HCT116 with PTPRS and PTPRS KO cells in response to 48 hours incubation with ERKi (VX11E and SCH772984) and MEKi (AZD8330, Trametinib, and PD0325). (**B**) Dose response for ERKi SCH772984 in cells with PTPRS and PTPRS KO cells treated 48 hours. (**C**) Dose response for PI3Ki LY294002 with and without ERKi SCH772984 (left). LY29002 did not generated a substantial amount of apoptosis in cells with PTPRS or without PTPRS. Combinations of LY29002 and SCH772984 showed an enhanced level of apoptosis. Enhanced apoptosis was also seen with AKTi and ERKi when used in combination (right). The paired, two-tailed *t* test was performed for comparisons. NS – not significant; ^*^ *p* < 0.05; ^**^ *p* < 0.01; ^***^ *p* < 0.001.

Data presented here and previously [18] show that the loss of PTPRS activity brought about increased ERK phosphorylation along with increased AKT phosphorylation. Since the increased ERK and AKT activity correlated with increased growth potential (Figure 1) we sought to determine if the increased ERK and AKT activities brought about a change in resistance to ERK and MEK inhibitors. In order to explore the induction of apoptosis and cell death we treated the HCT116 KRAS cell line pairs (+/– PTPRS) with inhibitors of ERK and measured annexin V staining. As previously shown [18], the PTPRS knockout cells had increased ERK and AKT phosphorylation (Figure 2). Figure 4 shows that the inhibition of ERK in the PTPRS CRISPR knockout cells had a greater induction of apoptosis than cells containing PTPRS activity. In close agreement, the inhibition of MEK produced greater apoptosis in PTPRS knockout cells that those cells with PTPRS activity (Figure 4A). Thus, the loss of PTPRS brought about increased ERK activity, ERK addiction and a decrease in adaptive resistance. Furthermore, when ERK and AKT/PI3K were both inhibited, apoptosis was equal or greater than additive (Figure 4C).

Since the loss of PTPRS brought about increased apoptosis in response to the inhibitors of ERK/MEK in HCT116 cells we used siRNA to ERK to inhibit ERK activity (as seen in Figure 3A). Interesting, the use of siRNA to ERK had shown a greater decrease in ERK and AKT phosphorylation with than seen with ERKi. The data in Figure 3B demonstrated that cells without PTPRS, when treated with siRNA to ERK, displayed an elevated level of apoptosis which was greater than the cells with PTPRS. The level of apoptosis was equal to that seen with the use of inhibitors to both ERK and AKT (Figure 4). The amounts of apoptosis induced by siRNA to ERK was greater in the CRISPR PTPRS KO cells than in cells with WT PTPRS. These data show the loss of PTPRS activity triggered a loss of resistance to MEKi/ERKi.

### The role of SRC in resistance to MEK and ERK inhibition

**Figure 5:**
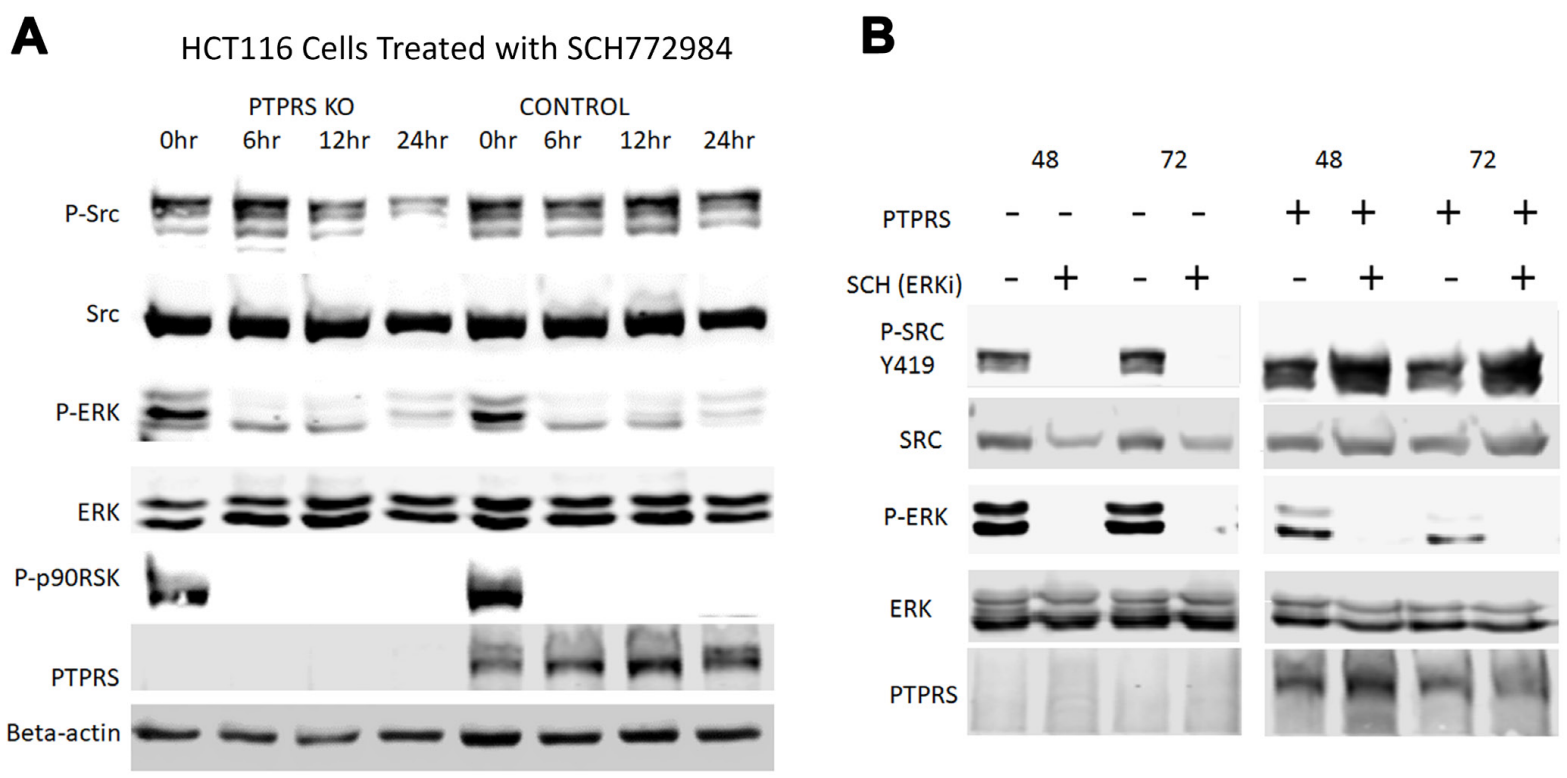
SRC activation in response to ERK inhibition is PTPRS dependent. Western blot analysis of HCT116 cells (+/– PTPRS) at various times after during ERKi. (**A**) HCT116 PTPRS KO and parental HCT116 cells (+PTPRS, mutant KRAS) were treated with 0.1 µM SCH772984 for 0, 6, 12, and 24 hours. The top row of lanes 1 through 4 revealed that phospho-SRC Y419 decreased in PTPRS KO cells when exposed to the ERKi for 24 hr hours (lane 4). Comparatively, phospho-SRC is maintained at a constant level in the PTPRS containing cells (lanes 5–8). (**B**) Comparison of the PTPRS KO cells to parental cells with PTPRS after incubation with ERKi for 48 hr (lanes 1 and 2) and 72 hours (lanes 3 and 4) to parental cells +PTPRS (lanes 5–8). The PTPRS KO cells treated with ERKi show a complete reduction in phospho-SRC after 48 hours, while the control cells showed increased phospho-SRC-Y419 levels. Each experiment was performed in triplicate with quantitative densitometry analysis performed (Supplementary Figure 4).

Even though the loss of PTPRS activity increased ERK and AKT activity, the loss of PTPRS also produced an increase in sensitivity to MEK/ERK inhibition, or an apparent decrease in the resistance to MEK/ERK inhibition. We had noticed that siRNA knockdown of PTPRS created a change in the cell morphology of growing CRC cells, and this change appeared as a more rounded, epithelial appearance (Supplementary Figure 1). Reduction of SRC activity has been shown to increase epithelial appearance [32–34], and since SRC was recently suggested to support adaptive resistance (AR) in ovarian cancer cell lines treated with MEK inhibitors, we sought to investigate a role for SRC activity in the increased sensitivity to ERKi in cells without PTPRS activity. In order to explore the possible effects of ERK/MEK inhibition on SRC activity, we treated the matched pair of isogenic CRC cell lines (+/– PTPRS) with an ERK inhibitor and followed SRC-Y419 phosphorylation during early and extended drug treatment. As seen in Figure 5A, the cells with PTPRS showed an early but slight decrease in SRC phosphorylation on Y-419, followed by a sizable, rapid, sustained increase in SRC activation continuing through 72 hours (Figure 5B). This early and sustained SRC elevation was consistent with an adaptive response. On the other hand, when cells without PTPRS (KO) were treated with an ERK inhibitor, a significant decrease in SRC activity was noted and was sustained for the duration of the treatment (72 hrs). In Figure 5B, the cells without PTPRS did not display phosphorylation of SRC after treatment with an ERKi for 48 or 72 hours. Cells with PTPRS had phosphorylated SRC and showed increased levels of SRC phosphorylation in response to ERKi. These data suggested that increased SRC activity may be responsible for resistance to MEK/ERK inhibition through the induction of an anti-apoptotic state or AR. CRC cells lacking PTPRS did not show an increase in SRC activation, and had a greater apoptotic response to ERK or MEK inhibition (Figure 4).

**Figure 6:**
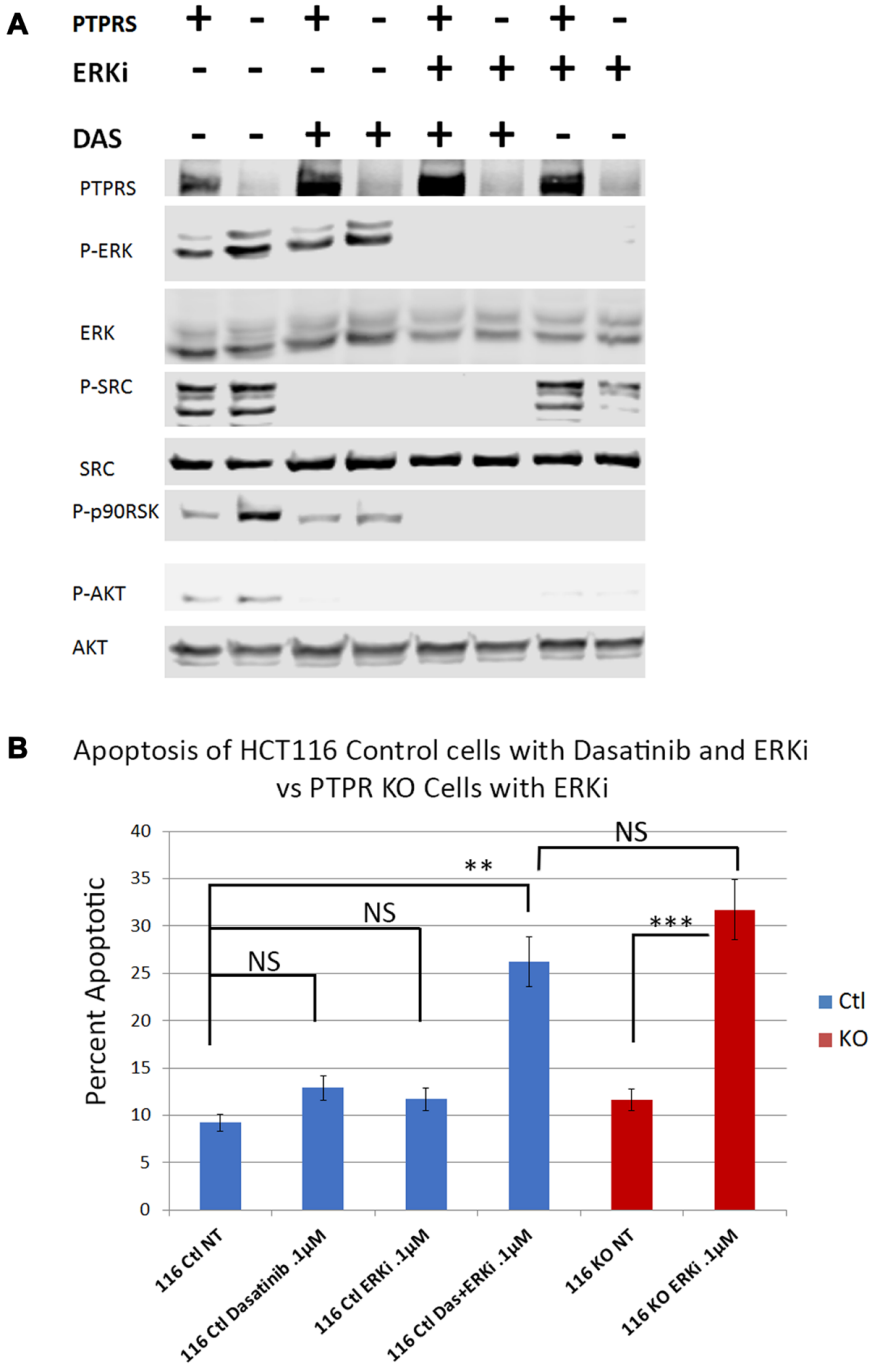
Dasatinib treatments increased apoptotic response to ERK inhibition in HCT116 cells. Parental cells with PTPRS (labeled PTPRS +) were treated with ERKi and Dasatinib for 48 hours and compared to PTPRS KO cells (labeled PTPRS -) which were treated with ERKi alone for 48 hours. (**A**) PTPRS containing cells (lanes PTPRS +) labeled: untreated, Dasatinib (0.1 µM), ERKi SCH772984 (0.1 µM), and a combination of Dasatinib and ERKi. The Dasatinib treatment showed a reduction in phospho-SRC-Y419, but little change in phospho-ERK1/2-T202/Y204. Conversely, the ERKi treatment decreased phospho-ERK1/2-T202/Y204 but not phospho-SRC-Y419. The combination of both inhibitors was able to eliminate the phosphorylation of both ERK and SRC. This phosphorylation pattern matches that of the PTPRS KO cells when treated solely with the ERKi. Lane 8 shows the decrease in phosph-SRC in PTPRS KO cells treated with ERKi. The experiment was performed in triplicate for quantitative densitometry analysis see Supplementary Figure 5. (**B**) when both Dasatinib and ERKi were used in combination (lane 4), treatment of HCT116 + PTPRS revealed the induction of apoptosis equal to that induced by ERKi *alone* in PTPRS KO cells (lane 6). The paired, two-tailed *t* test was performed for comparisons. NS – not significant; ^**^ *p* < 0.01; ^***^ *p* < 0.001.

**Figure 7:**
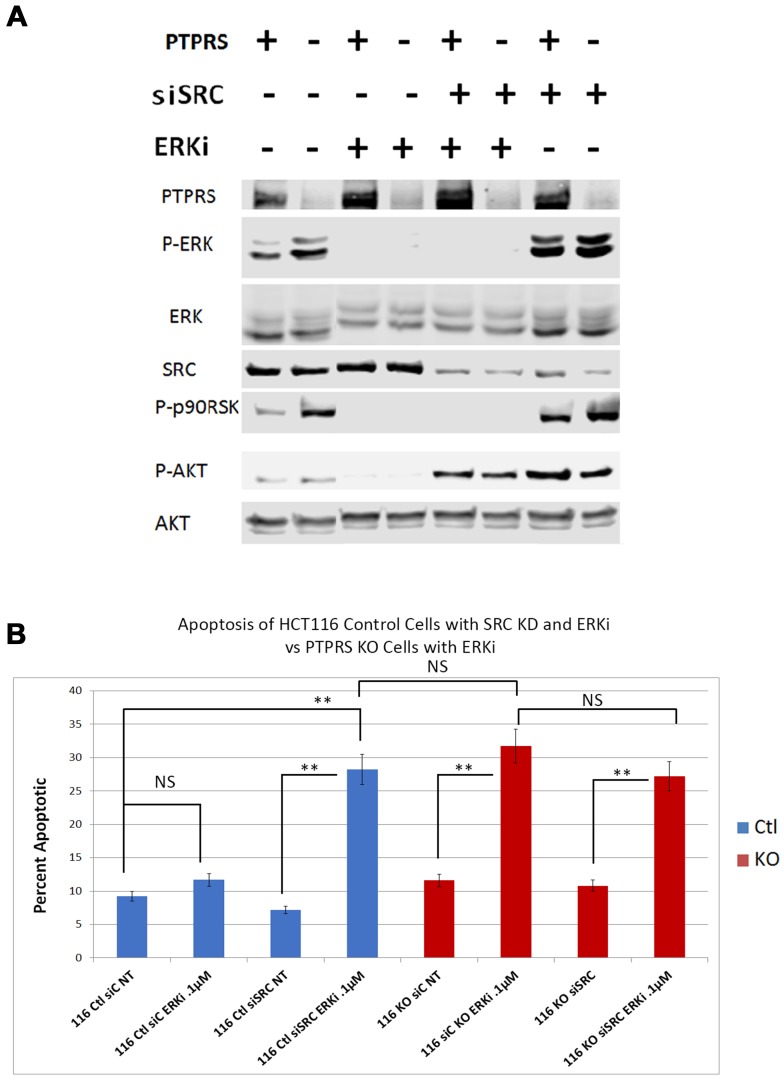
siRNA knockdown of SRC increased apoptotic response to ERK inhibition. HCT116 PTPRS KO and parental cells + PTPRS were treated with siRNA to SRC for 24 hours and then incubated with an ERKi for 48 hours. (**A**) Western analysis: Parental control cells (+PTPRS) treated with control siRNA (–), ERKi (SCH772984), siRNA to SRC (+), and a combination of SRC siRNA and ERKi; PTPRS KO (-PTPRS) cells were given the same treatments respectively. In the figure the two control lanes on the left were taken from the right end of this original blot and moved to conform to Figure 6 and for ease of understanding. The experiment was performed in triplicate for quantitative densitometry analysis see Supplementary Figure 6. (**B**) Annexin V apoptosis assays for these treatments revealed that the siRNA knockdown of SRC in combination with ERKi treatment in cells containing PTPRS caused the same amount of apoptosis as the ERKi in PTPRS KO cells. “116Ctl” represent parental cells + PTPRS, 116KO are HCT116 with PTPRS knockout cells, siC is siRNA scrambled control RNA, NT = not treated and ERKi = SCH 772984. The addition of the SRC siRNA 24 hours before a 48-hour ERK inhibition of the PTPRS KO cells did not enhance apoptosis (lane 6 compared to lane 8). The paired, two-tailed *t* test was performed for comparisons. NS – not significant; ^**^ *p* < 0.01.

In order to test the hypothesis that SRC activity was responsible for MEKi/ERKi resistance, we treated isogenic CRC cell lines +/– PTPRS with an ERK inhibitor in the presence or absence of dasatinib, an inhibitor of SRC. Figure 6 shows that dasatinib inhibited the SRC phosphorylation of Y-419, and enhanced the apoptosis induced by ERK inhibition. The level of apoptosis found in the cells containing PTPRS treated with ERKi and dasatinib was equal to the amount of apoptosis in cells with PTPRS knocked out and treated with ERKi (Figure 6B). These data suggest that CRC cells show resistance to MAPK pathway inhibitors---possibly induced via elevated SRC activity---and that the inhibition of SRC activity produced an increased apoptotic response to ERKi. To verify the specificity of the SRC inhibition for the increased apoptosis observed after ERKi, we used siRNA to SRC to specifically remove SRC. When cells were treated with the siRNA to SRC, as seen in Figure 7, the total SRC was substantially reduced. We treated CRC cells expressing PTPRS with an ERKi following the use of siRNA to knockdown SRC and found these cells had a significant apoptotic response to ERKi, which was similar to that observed in cells with KO PTPRS treated with an ERKi. Thus, SRC knock-down (siRNA) or SRC inhibition (dasatinib) allowed an increased apoptosis response to ERKi in PTPRS containing cells which was equal to the apoptosis found in cells with KO PTPRS treated with an ERKi (Figures 6 and 7).

## DISCUSSION

Using an integrated approach to compare gene mutations with global gene expression in our 468 human CRC tumor collection, we showed that mutations in PTPRS highly correlated with an increase in a validated RAS pathway activity signature [18, 19]. We found that a range of native PTPRS missense mutations in CRC had detrimental effects on its phosphatase activity directed at ERK [18]. Thus, the numerous mutations we discovered affecting multiple PTPRS functional protein domains could alter its activity. We have shown that the loss of PTPRS activity in CRC cell lines produced an increase in ERK and AKT activation [18] and this increase was independent of mutationally-activated KRAS. Not surprisingly, the increase in ERK and AKT activation brought about an expected increase in growth potential (Figure 1). We also observed that the increase in AKT activation found in CRC cells with KO PTPRS was dependent on the presence of ERK activity (Figure 2). It is unclear at present why ERK activity is necessary for the increase and/or stability of AKT phosphorylation. Possible crosstalk between ERK and AKT has previously been explored by others [35–39].

The loss of PTPRS not only brought about increased ERK and AKT activity, but we were surprised to find that the CRC cells without PTPRS activity, despite the increased ERK activity, became more sensitive to ERK inhibition as was seen in Figure 4. The HCT116 PTPRS KO cells with elevated ERK activity had increased apoptosis when treated with an ERKi. We also note that these cells were also more sensitive to MEK inhibition. From our data we propose that drug resistance may represent an apoptotic by-pass pathway [19], and/or an adaptive resistance mechanism [16, 40, 41] which is dependent on PTPRS activity. Recently, SRC activity was suggested to play a role in adaptive resistance when ovarian cancer cell lines were treated with a MEK inhibitor [16]. Interestingly, it is well established that many CRC tumors, particularly those with advanced stage or metastasis, have a higher level of activated SRC than normal colon cells. Thus, we decided to examine SRC-Y419 phosphorylation in the CRC cells with or without PTPRS after ERK inhibition. Even though the cell lines had similar amounts of tyrosine-419 phospho-SRC before ERK inhibition, they responded differently to the ERKi. While cells with PTPRS showed an increase in SRC-Y419 phosphorylation in the presents of ERKi, the cells without PTPRS activity showed marked continued decrease in SRC phosphorylation during ERK inhibition. Thus, our data describe an apparent adaptive resistance to MEKi/ERKi that requires the presence of PTPRS. Similar to investigations reported from the laboratory of Slingerland [16], we show that adaptive resistance to MEK/ERK inhibition could be through SRC activation (Figure 5). We tested our hypothesis by inhibiting SRC activity using dasatanib in parental CRC cell lines with PTPRS and then treating those cells with ERKi. The inhibition of SRC increased ERKi induced apoptosis in cells with PTPRS activity to the same level found in cells with KO PTPRS treated with ERKi (Figure 6). In order to insure the specificity related to SRC we knocked-down SRC with specific siRNA. Our data show that ERKi treatment along with siRNA to SRC in cells expressing PTPRS mimicked the response seen in cells with KO PTPRS treated with ERKi (Figure 7). These data with siRNA to SRC agree with our findings of ERKi induced apoptosis in the presence of the SRC inhibitor dasatinib. The increase in SRC activity after ERKi explains what appears to be an adaptive resistance response, with the potential to avert it using the SRC-specific inhibitor, dasatinib. Thus, the loss of PTPRS may not induce an increase in sensitivity, but rather produces a *decrease in resistance*. The lack of SRC activity in cells without PTPRS was thought possibly due to increased phosphorylation of Y-530 which would negatively regulate SRC activity [15] and explain how PTPRS promoted an increase in SRC activity. However, +/– PTPRS did not bring about a detectable change in the phosphorylation of Y-530, suggesting other potential mechanisms. Our data and another recent publication [16] show that a combination of a MEKi or ERKi with a SRCi may prove to be a useful therapeutic regimen for colorectal cancers by not allowing MEKi or ERKi induced adaptive resistance, increasing the potential utility of these novel drugs. Furthermore, our data support our hypothesis that PTPRS induces or maintains resistance to MEKi/ERKi by an increase in SRC activation.

## MATERIALS AND METHODS

### Cell culture

The parental HCT116 CRC cell line (KRASG13D/+) and the engineered HCT116-WT KRAS cell line, were obtained from Horizon Discovery (Cat.No.HD104-008). All cell lines had monthly tests for mycoplasma contamination with Sigma LookOut^®^ Mycoplasma qPCR Detection Kit (Cat No. MP0040A-1KT). Cells were cultured using RPMI 1640 (Gibco) supplemented with 10% FBS and 1% penicillin and streptomycin, unless otherwise specified.

### Immunoblotting

Cell lysates were obtained using 1x RIPA buffer (9806 Cell Signaling) containing 10 mM PMSF, Protease Inhibitor Cocktail (M250 Amresco), Phosphatase Inhibitor Cocktail 2 (P5726 Millipore), and Phosphatase Inhibitor Cocktail 3 (P0044 Millipore). The LI-COR Odyssey^®^ CLx Imaging System was used to image all immunoblots. Antibodies were typically duplexed using rabbit antibodies for phosphorylated antibodies and mouse antibodies for total protein. Li-Cor secondary antibodies, Goat anti-Rabbit IRDye 680RD and Goat anti-Mouse IRDye 800CW, were used with the duplexed primary antibodies.

Rabbit primary antibodies were used unless specified and were sourced as follows: PTPRS (goat AF3430 R&D Systems); alpha-Tubulin (mouse sc-8035 Santa Cruz). All other antibodies were obtained from Cell Signaling: phospho-Erk1/2 T202/Y204 (Cat.No.4370); phosphor-Akt S473 (mouse 4051); Akt (4691); phosphor-src Y416 (2101) and Src (mouse 2105).

### siRNA transfection

Two PTPRS-specific siRNAs were obtained from Qiagen: PTPRS_5 siRNA (SI02759288 Qiagen, target sequence: CAGGACATTCTCTCTGCACAA); PTPRS_7 siRNA (SI03056284 Qiagen, target sequence: ATGGCGTGCCCGAATACCCAA).

Two SRC siRNA duplexes were obtained from Origene:

SR321884A: GGUUGUAAAUACUUUGCAUAUUGTC

SR321884B: GCAAGGUGCCAAAUUCCCCAUCAAG

Scrambled siRNAs from Qiagen (SI03650325) and Origene (SR30004) were used as controls. Transfections were performed at 20–30% cell confluency using the RNAiMAX Lipofectamine (Life Tech) according to the provided protocol using 30 nM of siRNA.

### CRISPR knockout of PTPRS

The CRISPR kit for PTPRS was purchased from Origene (Cat.No.KN211163) and used according to the product protocol. Cells were transfected using Lipofectamine3000. The gRNA sequence KN211163G1, PTPRS gRNA vector 1 in pCas-Guide vector, (target sequence: CTTGTGGTCCTGCTCGTTGG) proved the most effective at knocking out (KO) PTPRS expression and was thus used to create the HCT116 and HCT116-KRAS (–/+) PTPRS KO cell lines. CRISPR cells were then grown for 7 passages and selected using puromycin (Life Technologies). Numerous colonies were isolated and tested for absence of PTPRS via Western blot and mRNA analysis.

### Inhibitors and apoptosis assay

HCT116 PTPRS KO Cells and control cells were treated with the ERK inhibitors SCH772984 (942183-80-4 Santa Cruz Biotechnology), VX11-E (A3931 ApexBio), VRT752271 (B1106 ApexBio); MEK inhibitors Trametinib (A3018 ApexBio) and AZD8330 (A8374 ApexBio); PI3K inhibitor LY294002 (Cell Signaling 9901), Dasatinib (CDS023389 Sigma) and the AKT inhibitor VIII (A6730 Sigma). All treatments were for 48 hours and the noted concentrations, unless specified otherwise. Following treatments, Annexin 5 apoptosis assay or a cell cycle analysis using Brdu and propidium iodide staining were performed as described previously [42]. The analyses were performed with a BD Accuri-C6 Flow Cytometer.

### Statistical analysis

Cell culture experiments were done in triplicates, and mean and standard deviation were calculated as indicated. Two-tailed, paired *t* test was used to determine the statistical significance of comparison as needed. All Western blot experiments were performed in triplicate. Quantitative analysis was performed with Licor Image Studio 5.2 (Licor). Band density was determined via median analysis on the “all” setting. Numbers from each blot were normalized by dividing phosphorylation signal by total protein; then averaging the values of all three experiments (see Supplementary Figures 2–6).

## SUPPLEMENTARY MATERIALS



## References

[1] Siegel RL , Miller KD , Jemal A . Cancer statistics, 2016. CA Cancer J Clin. 2016; 66:7–30. 10.3322/caac.21332. 26742998

[2] Cancer Genome Atlas Network. Comprehensive molecular characterization of human colon and rectal cancer. Nature. 2012; 487:330–7. 10.1038/nature11252. 22810696 PMC3401966

[3] Schubbert S , Shannon K , Bollag G . Hyperactive Ras in developmental disorders and cancer. Nat Rev Cancer. 2007; 7:295–308. 10.1038/nrc2109. 17384584

[4] Schell MJ , Yang M , Teer JK , Lo FY , Madan A , Coppola D , Monteiro AN , Nebozhyn MV , Yue B , Loboda A , Bien-Willner GA , Greenawalt DM , Yeatman TJ . A multigene mutation classification of 468 colorectal cancers reveals a prognostic role for APC. Nat Commun. 2016; 7:11743. 10.1038/ncomms11743. 27302369 PMC4912618

[5] Giannakis M , Mu XJ , Shukla SA , Qian ZR , Cohen O , Nishihara R , Bahl S , Cao Y , Amin-Mansour A , Yamauchi M , Sukawa Y , Stewart C , Rosenberg M , et al. Genomic Correlates of Immune-Cell Infiltrates in Colorectal Carcinoma. Cell Rep. 2016; 17:1206. 10.1016/j.celrep.2016.10.009. 27760322 PMC5638785

[6] Popovici V , Budinska E , Tejpar S , Weinrich S , Estrella H , Hodgson G , Van Cutsem E , Xie T , Bosman FT , Roth AD , Delorenzi M . Identification of a poor-prognosis BRAF-mutant-like population of patients with colon cancer. J Clin Oncol. 2012; 30:1288–95. 10.1200/JCO.2011.39.5814. 22393095

[7] Schell MJ , Yang M , Missiaglia E , Delorenzi M , Soneson C , Yue B , Nebozhyn MV , Loboda A , Bloom G , Yeatman TJ . A Composite Gene Expression Signature Optimizes Prediction of Colorectal Cancer Metastasis and Outcome. Clin Cancer Res. 2016; 22:734–45. 10.1158/1078-0432.CCR-15-0143. 26446941 PMC4802496

[8] Amaral T , Sinnberg T , Meier F , Krepler C , Levesque M , Niessner H , Garbe C . MAPK pathway in melanoma part II-secondary and adaptive resistance mechanisms to BRAF inhibition. Eur J Cancer. 2017; 73:93–101. 10.1016/j.ejca.2016.12.012. 28162869

[9] Chandarlapaty S . Negative feedback and adaptive resistance to the targeted therapy of cancer. Cancer Discov. 2012; 2:311–9. 10.1158/2159-8290.CD-12-0018. 22576208 PMC3351275

[10] Fedele C , Ran H , Diskin B , Wei W , Jen J , Geer MJ , Araki K , Ozerdem U , Simeone DM , Miller G , Neel BG , Tang KH . SHP2 Inhibition Prevents Adaptive Resistance to MEK Inhibitors in Multiple Cancer Models. Cancer Discov. 2018; 8:1237–49. 10.1158/2159-8290.CD-18-0444. 30045908 PMC6170706

[11] Ma P , Fu Y , Chen M , Jing Y , Wu J , Li K , Shen Y , Gao JX , Wang M , Zhao X , Zhuang G . Adaptive and Acquired Resistance to EGFR Inhibitors Converge on the MAPK Pathway. Theranostics. 2016; 6:1232–43. 10.7150/thno.14409. 27279914 PMC4893648

[12] Pazarentzos E , Bivona TG . Adaptive stress signaling in targeted cancer therapy resistance. Oncogene. 2015; 34:5599–606. 10.1038/onc.2015.26. 25703329 PMC4546915

[13] Rosell R , Karachaliou N , Morales-Espinosa D , Costa C , Molina MA , Sansano I , Gasco A , Viteri S , Massuti B , Wei J , Gonzalez Cao M , Martinez Bueno A . Adaptive resistance to targeted therapies in cancer. Transl Lung Cancer Res. 2013; 2:152–9. 10.3978/j.issn.2218-6751.2012.12.08. 25806228 PMC4367602

[14] Lieu C , Kopetz S . The SRC family of protein tyrosine kinases: a new and promising target for colorectal cancer therapy. Clin Colorectal Cancer. 2010; 9:89–94. 10.3816/CCC.2010.n.012. 20378502 PMC3091503

[15] Yeatman TJ . A renaissance for SRC. Nat Rev Cancer. 2004; 4:470–80. 10.1038/nrc1366. 15170449

[16] Simpkins F , Jang K , Yoon H , Hew KE , Kim M , Azzam DJ , Sun J , Zhao D , Ince TA , Liu W , Guo W , Wei Z , Zhang G , et al. Dual Src and MEK Inhibition Decreases Ovarian Cancer Growth and Targets Tumor Initiating Stem-Like Cells. Clin Cancer Res. 2018; 24:4874–86. 10.1158/1078-0432.CCR-17-3697. 29959144 PMC6557165

[17] Cordero JB , Ridgway RA , Valeri N , Nixon C , Frame MC , Muller WJ , Vidal M , Sansom OJ . c-Src drives intestinal regeneration and transformation. EMBO J. 2014; 33:1474–91. 10.1002/embj.201387454. 24788409 PMC4194090

[18] Davis TB , Yang M , Schell MJ , Wang H , Ma L , Pledger WJ , Yeatman TJ . PTPRS Regulates Colorectal Cancer RAS Pathway Activity by Inactivating Erk and Preventing Its Nuclear Translocation. Sci Rep. 2018; 8:9296. 10.1038/s41598-018-27584-x. 29915291 PMC6006154

[19] Dry JR , Pavey S , Pratilas CA , Harbron C , Runswick S , Hodgson D , Chresta C , McCormack R , Byrne N , Cockerill M , Graham A , Beran G , Cassidy A , et al. Transcriptional pathway signatures predict MEK addiction and response to selumetinib (AZD6244). Cancer Res. 2010; 70:2264–73. 10.1158/0008-5472.can-09-1577. 20215513 PMC3166660

[20] Omolo B , Yang M , Lo FY , Schell MJ , Austin S , Howard K , Madan A , Yeatman TJ . Adaptation of a RAS pathway activation signature from FF to FFPE tissues in colorectal cancer. BMC Med Genomics. 2016; 9:65. 10.1186/s12920-016-0225-2. 27756306 PMC5069826

[21] Elchebly M , Wagner J , Kennedy TE , Lanctot C , Michaliszyn E , Itie A , Drouin J , Tremblay ML . Neuroendocrine dysplasia in mice lacking protein tyrosine phosphatase sigma. Nat Genet. 1999; 21:330–3. 10.1038/6859. 10080191

[22] Shen Y , Tenney AP , Busch SA , Horn KP , Cuascut FX , Liu K , He Z , Silver J , Flanagan JG . PTPsigma is a receptor for chondroitin sulfate proteoglycan, an inhibitor of neural regeneration. Science. 2009; 326:592–6. 10.1126/science.1178310. 19833921 PMC2811318

[23] Wallace MJ , Batt J , Fladd CA , Henderson JT , Skarnes W , Rotin D . Neuronal defects and posterior pituitary hypoplasia in mice lacking the receptor tyrosine phosphatase PTPsigma. Nat Genet. 1999; 21:334–8. 10.1038/6866. 10080192

[24] Muise AM , Walters T , Wine E , Griffiths AM , Turner D , Duerr RH , Regueiro MD , Ngan BY , Xu W , Sherman PM , Silverberg MS , Rotin D . Protein-tyrosine phosphatase sigma is associated with ulcerative colitis. Curr Biol. 2007; 17:1212–8. 10.1016/j.cub.2007.06.013. 17614280

[25] Martin KR , Xu Y , Looyenga BD , Davis RJ , Wu CL , Tremblay ML , Xu HE , MacKeigan JP . Identification of PTPsigma as an autophagic phosphatase. J Cell Sci. 2011; 124:812–9. 10.1242/jcs.080341. 21303930 PMC3039021

[26] Murchie R , Guo CH , Persaud A , Muise A , Rotin D . Protein tyrosine phosphatase sigma targets apical junction complex proteins in the intestine and regulates epithelial permeability. Proc Natl Acad Sci U S A. 2014; 111:693–8. 10.1073/pnas.1315017111. 24385580 PMC3896190

[27] MacKeigan JP , Murphy LO , Blenis J . Sensitized RNAi screen of human kinases and phosphatases identifies new regulators of apoptosis and chemoresistance. Nat Cell Biol. 2005; 7:591–600. 10.1038/ncb1258. 15864305

[28] Morris LG , Taylor BS , Bivona TG , Gong Y , Eng S , Brennan CW , Kaufman A , Kastenhuber ER , Banuchi VE , Singh B , Heguy A , Viale A , Mellinghoff IK , et al. Genomic dissection of the epidermal growth factor receptor (EGFR)/PI3K pathway reveals frequent deletion of the EGFR phosphatase PTPRS in head and neck cancers. Proc Natl Acad Sci U S A. 2011; 108:19024–9. 10.1073/pnas.1111963108. 22065749 PMC3223475

[29] Wang ZC , Gao Q , Shi JY , Guo WJ , Yang LX , Liu XY , Liu LZ , Ma LJ , Duan M , Zhao YJ , Wu YN , Gao DM , Wang XY , et al. Protein tyrosine phosphatase receptor S acts as a metastatic suppressor in hepatocellular carcinoma by control of epithermal growth factor receptor-induced epithelial-mesenchymal transition. Hepatology. 2015; 62:1201–14. 10.1002/hep.27911. 25998839

[30] Suarez Pestana E , Tenev T , Gross S , Stoyanov B , Ogata M , Bohmer FD . The transmembrane protein tyrosine phosphatase RPTPsigma modulates signaling of the epidermal growth factor receptor in A431 cells. Oncogene. 1999; 18:4069–79. 10.1038/sj.onc.1202794. 10435588

[31] Morris LG , Chan TA . Resistance to EGFR inhibitors: molecular determinants and the enigma of head and neck cancer. Oncotarget. 2011; 2:894–5. 10.18632/oncotarget.407. 22248868 PMC3282094

[32] Patel A , Sabbineni H , Clarke A , Somanath PR . Novel roles of Src in cancer cell epithelial-to-mesenchymal transition, vascular permeability, microinvasion and metastasis. Life Sci. 2016; 157:52–61. 10.1016/j.lfs.2016.05.036. 27245276 PMC4956571

[33] Thiery JP . Epithelial-mesenchymal transitions in tumour progression. Nat Rev Cancer. 2002; 2:442–54. 10.1038/nrc822. 12189386

[34] Irby RB , Yeatman TJ . Role of Src expression and activation in human cancer. Oncogene. 2000; 19:5636–42. 10.1038/sj.onc.1203912. 11114744

[35] Aksamitiene E , Kholodenko BN , Kolch W , Hoek JB , Kiyatkin A . PI3K/Akt-sensitive MEK-independent compensatory circuit of ERK activation in ER-positive PI3K-mutant T47D breast cancer cells. Cell Signal. 2010; 22:1369–78. 10.1016/j.cellsig.2010.05.006. 20471474 PMC2893265

[36] Dent P . Crosstalk between ERK, AKT, and cell survival. Cancer Biol Ther. 2014; 15:245–6. 10.4161/cbt.27541. 24424114 PMC3974823

[37] Mendoza MC , Er EE , Blenis J . The Ras-ERK and PI3K-mTOR pathways: cross-talk and compensation. Trends Biochem Sci. 2011; 36:320–8. 10.1016/j.tibs.2011.03.006. 21531565 PMC3112285

[38] Sun X , Bao J , You Z , Chen X , Cui J . Modeling of signaling crosstalk-mediated drug resistance and its implications on drug combination. Oncotarget. 2016; 7:63995–4006. 10.18632/oncotarget.11745. 27590512 PMC5325420

[39] Toulany M , Minjgee M , Saki M , Holler M , Meier F , Eicheler W , Rodemann HP . ERK2-dependent reactivation of Akt mediates the limited response of tumor cells with constitutive K-RAS activity to PI3K inhibition. Cancer Biol Ther. 2014; 15:317–28. 10.4161/cbt.27311. 24351425 PMC3974833

[40] Caunt CJ , Sale MJ , Smith PD , Cook SJ . MEK1 and MEK2 inhibitors and cancer therapy: the long and winding road. Nat Rev Cancer. 2015; 15:577–92. 10.1038/nrc4000. 26399658

[41] Lito P , Rosen N , Solit DB . Tumor adaptation and resistance to RAF inhibitors. Nat Med. 2013; 19:1401–9. 10.1038/nm.3392. 24202393

[42] Fu W , Sharma SS , Ma L , Chu B , Bui MM , Reed D , Pledger WJ . Apoptosis of osteosarcoma cultures by the combination of the cyclin-dependent kinase inhibitor SCH727965 and a heat shock protein 90 inhibitor. Cell Death Dis. 2013; 4:e566. 10.1038/cddis.2013.101. 23538447 PMC3613821

