# Supplementary material for: PTPRS drives adaptive resistance to MEK/ERK inhibitors through SRC
Source: Oncotarget. 2019 Nov 26;10(63):6768–80. doi: 10.18632/oncotarget.27335 (PMC6887575; doi:10.18632/oncotarget.27335)
Supplement: Supplementary file 1 [file oncotarget-10-6768-s001.pdf]

PTPRS drives adaptive resistance to MEK/ERK inhibitors through SRC

SUPPLEMENTARY MATERIALS

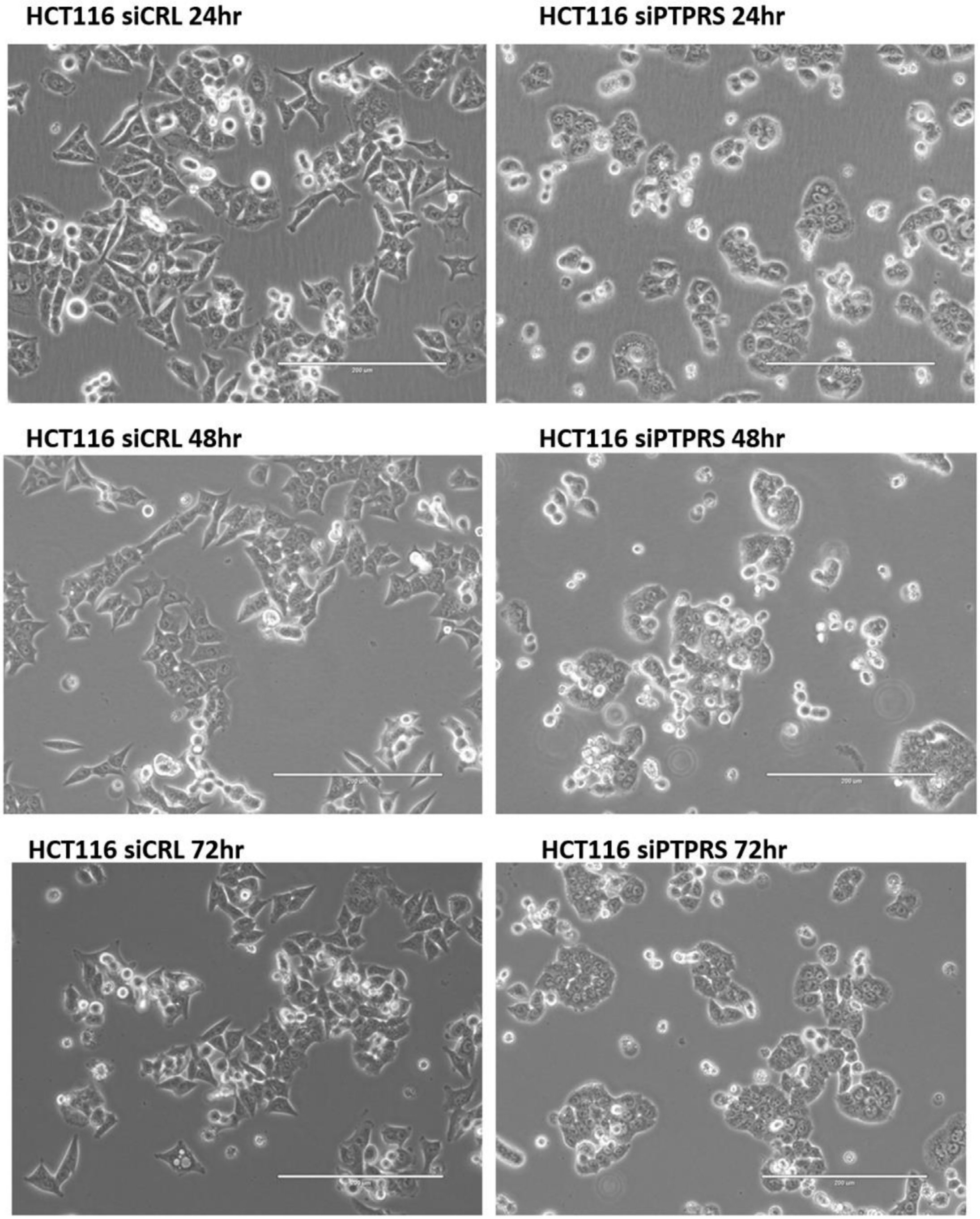

Supplementary Figure 1: siRNA knockdown of PTPRS in HCT116 cells produced an epithelial morphology. Via a transient knockdown of PTPRS, cell morphology appeared more epithelial like. Parental type HCT116 cells were treated with a scrambled control (left column) or a PTPRS targeted siRNA (right column), and were photographed at 24 hr, 48 hr, and 72 hr (as shown). The rounded look of the cells (vs. a spindle shape) with the PTPRS knockdown suggested an epithelial morphology.

Figure 2A Densitometry Values

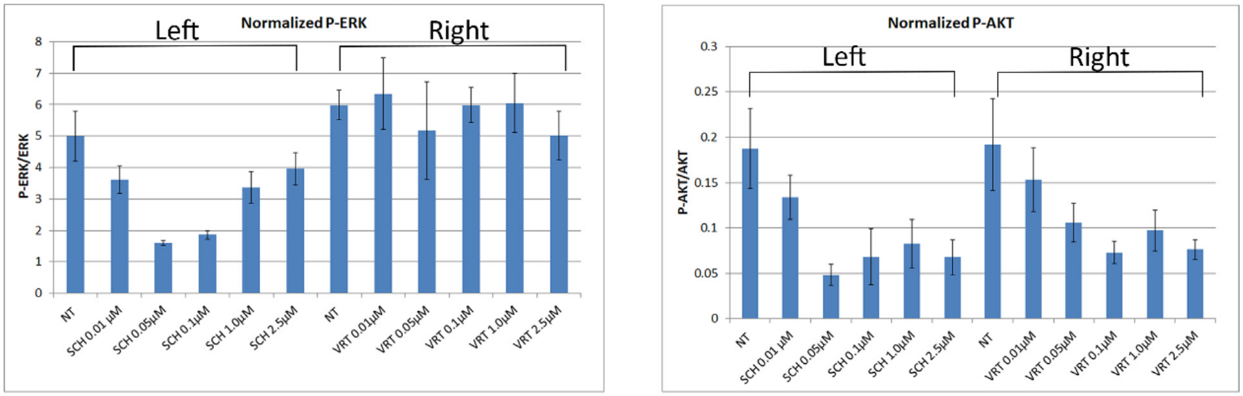

Figure 2B Densitometry Values

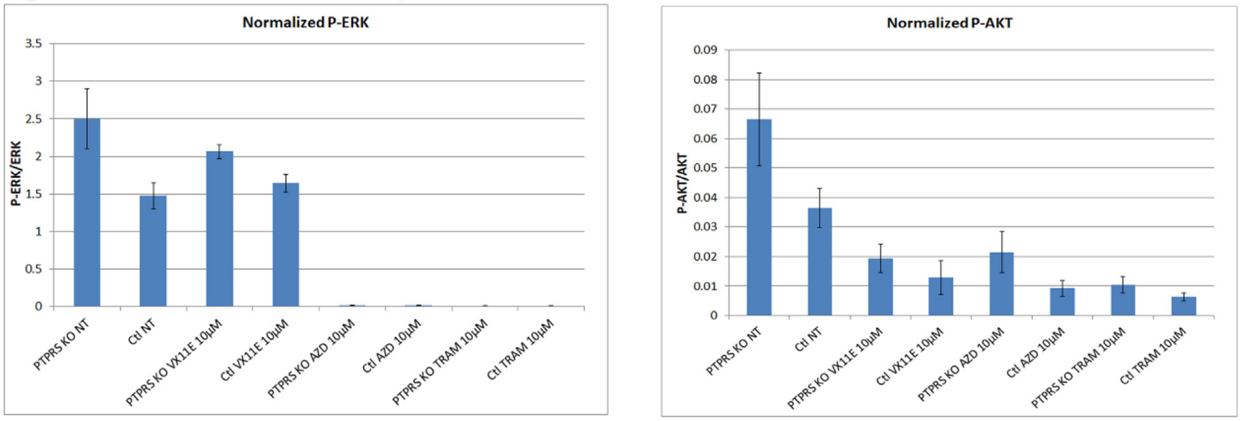

Figure 2C Densitometry Values

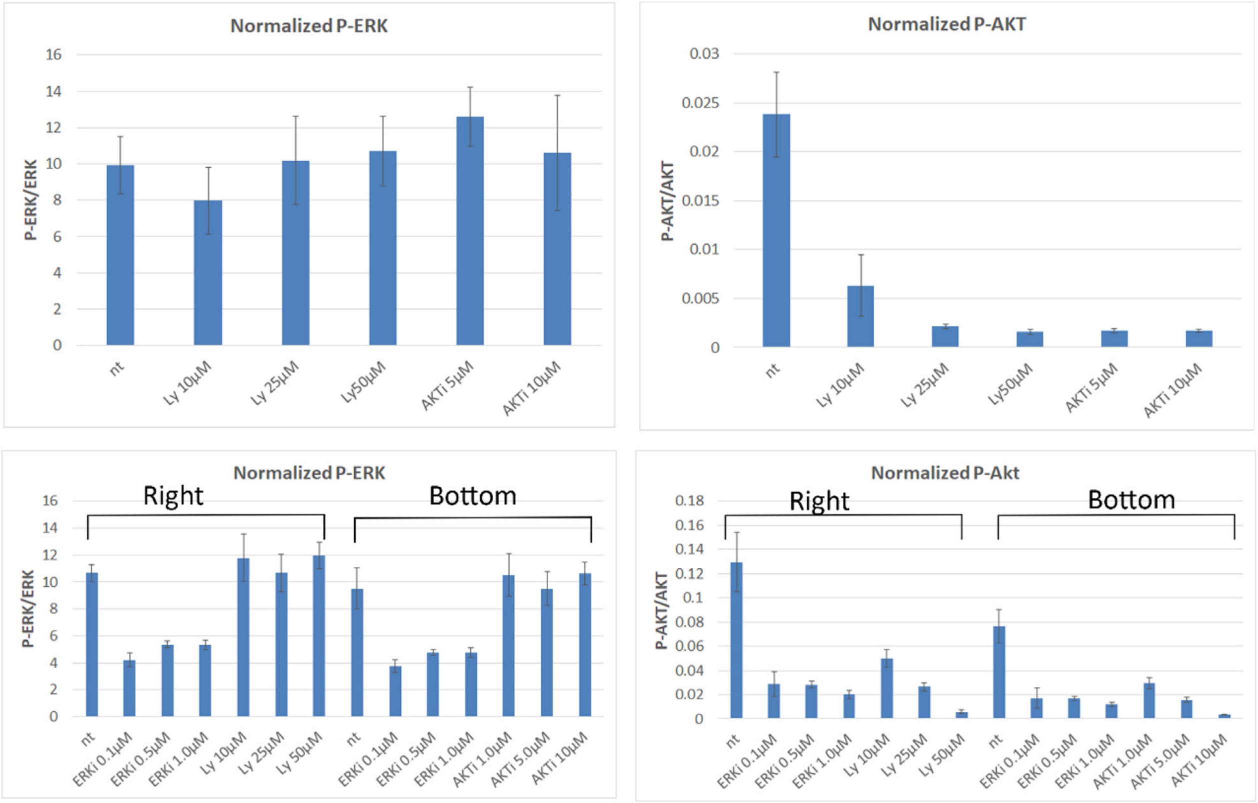

Supplementary Figure 2: Quantitative densitometry analysis of Western blots presented in Figure 2. Each Western blot was performed in triplicate. Error bars represent standard deviation.

Figure 3A Densitometry Values

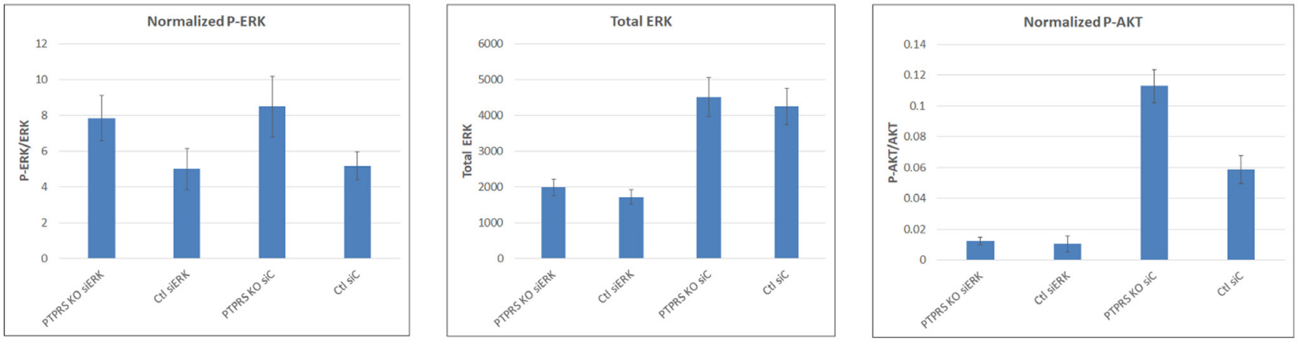

Supplementary Figure 3: Quantitative densitometry analysis of Western blots presented in Figure 3. Each Western blot was performed in triplicate. Error bars represent standard deviation.

Figure 5A Densitometry Values

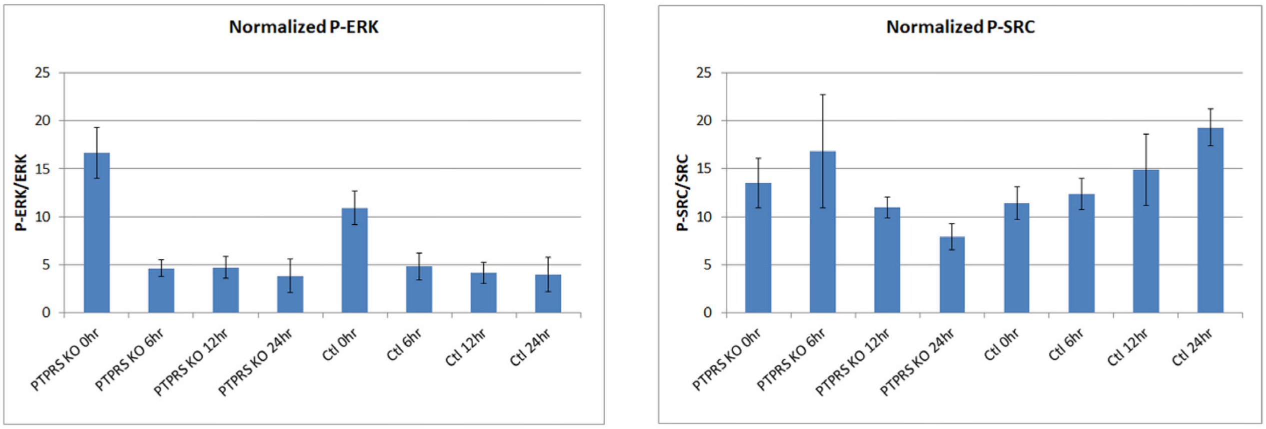

Figure 5B Densitometry Values

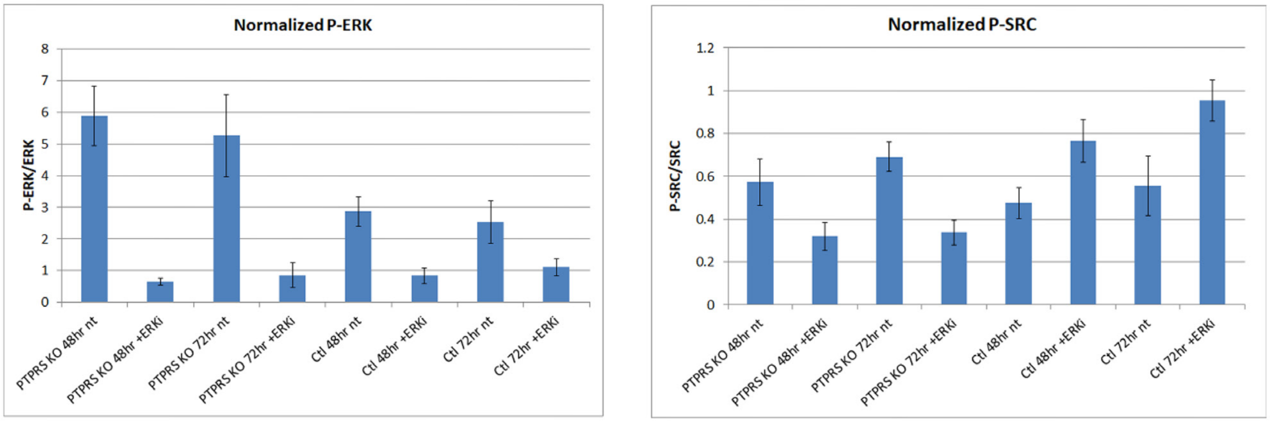

Supplementary Figure 4: Quantitative densitometry analysis of Western blots presented in Figure 5. Each Western blot was performed in triplicate. Error bars represent standard deviation.

Figure 6 Densitometry Values

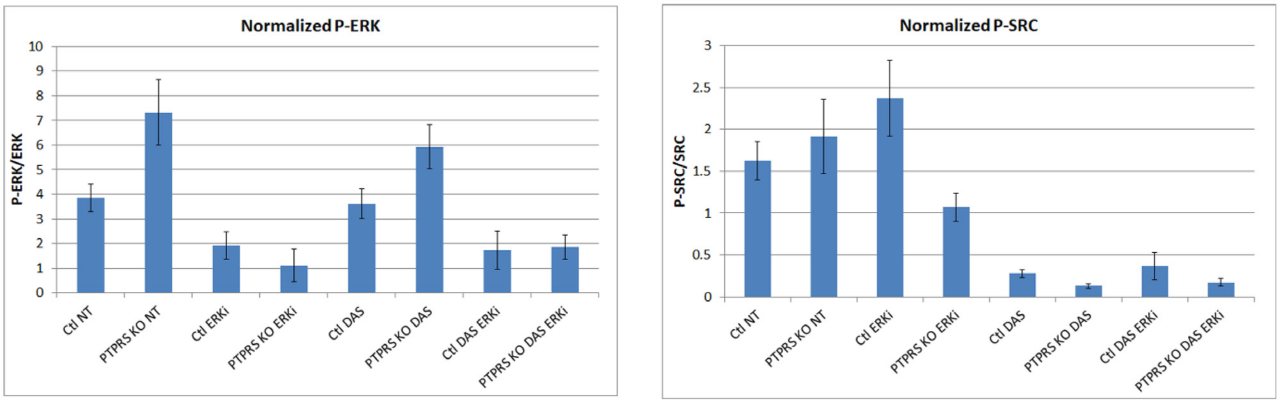

Supplementary Figure 5: Quantitative densitometry analysis of Western blots presented in Figure 6. Each Western blot was performed in triplicate. Error bars represent standard deviation.

Figure 7 Densitometry Values

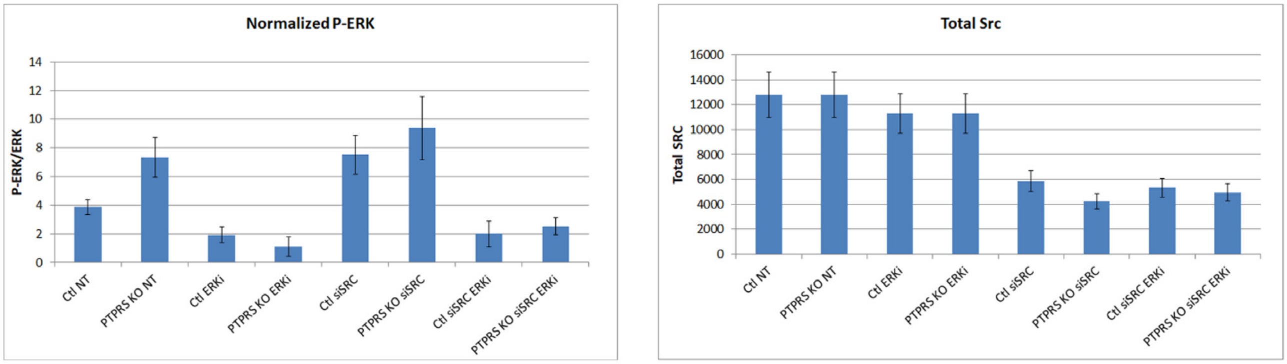

Supplementary Figure 6: Quantitative densitometry analysis of Western blots presented in Figure 7. Each Western blot was performed in triplicate. Error bars represent standard deviation.
